# **Pregnancy with autoimmune hepatitis**

**Published:** 2016

**Authors:** António Costa Braga, Carlos Vasconcelos, Jorge Braga

**Affiliations:** 1 *Obstetrics Department, Centro Materno Infantil do Norte - Centro Hospitalar do Porto (CHP), Oporto, Portugal *; 2 *Clinical Immunology Unit. CHP, Oporto, Portugal*

**Keywords:** high-risk pregnancy, liver disorder and pregnancy, azathioprine

## Abstract

**Aim:**

The aim of this study was to review our experience with gestations in autoimmune hepatitis patients.

**Background:**

There are only limited data describing pregnancy in patients with autoimmune hepatitis.

**Patients and methods:**

Retrospective analysis of pregnancies with autoimmune hepatitis followed in Centro Hospitalar do Porto, Portugal in the last ten years.

**Results:**

We reported nine pregnancies in seven patients with autoimmune hepatitis. Two patients had documented liver cirrhosis prior to the pregnancy. In this study, 66.7% of patients were treated with azathioprine and 88.9% with prednisolone. Clinical improvements were observed in 11.1% of pregnancies and 22.2% exacerbations were diagnosed. There were six live births and two preterm deliveries (preterm delivery rate of 33%). We also report three first trimester miscarriages (early gestation miscarriage rate of 33%). There were no neonatal or maternal deaths.

**Conclusion:**

The favorable obstetric outcome is a realistic expectation in patients with autoimmune hepatitis. Tight monitoring and control of asymptomatic and unpredictable exacerbations, which are unrelated to the severity of the underlying disease, are essential to the prognosis of the current pregnancy.

## Introduction

 Autoimmune hepatitis (AIH) is an autoimmune liver disorder described in 1953 (1). This pathology is characterized by a progressive hepatocellular inflammation that cannot be explained by alcohol consumption, viral infection, exposure to hepatotoxic drugs, or genetic liver disorders (2-5). AIH usually affects young women at childbearing age (2). In spite of the frequently associated oligomenorrhea (6), the number of pregnancies described in these patients are becoming more common as a result of the better clinical management (3). Optimal management during pregnancy is not yet well defined (7), and flares occur in 7 to 33% of pregnancies (7-11) and 11 to 86% postpartum (7-11). The majority of these flares can be controlled with an increment of immunosuppression (7-11). However, severe flares with hepatic decompensation have been described (10). 

Pregnancy in these patients is associated with a higher than normal rate of miscarriage, stillbirth, and premature delivery (5,7-13). In the presence of portal hypertension with oesophageal varices, the risk of bleeding is higher during pregnancy, particularly in the second half of gestation (14). Fetal outcomes in pregnancies with AIH are good and similar to other autoimmune disorders (10) with live birth rates being superior to 70% in the recent series (7-11). The aim of this study was to review our experience with gestations in AIH patients. 

## Patients and Methods

We conducted a retrospective descriptive study that included all pregnant patients with a diagnosis of AIH that were followed in the Obstetric Department of Centro Hospitalar do Porto, Oporto, Portugal, between 2004 and 2014. Seven patients with a total of nine pregnancies were followed. The course of gestation, delivery and puerperium were analyzed retrospectively, and data was collected from patients’ medical records. The diagnosis of AIH was established by our Internal Medicine/Hepatology department based on IAHG criteria (15). Cirrhotic patients were identified according to the presence of previous histologic findings.

All patients were followed by a multidisciplinary team, constituted by hepathologists and high-risk obstetricians. Antenatal visits were scheduled once a month up to 28 weeks of pregnancy, every two weeks up to 32 weeks, and thereafter weekly. A pre-conceptional liver function evaluation was performed in all cases and periodic hepatic evaluation was performed with monthly analytical evaluation. In patients with hepatic cirrhosis and portal hypertension, oesophageal varices evaluation was performed during the second trimester and prophylactic treatment applied when indicated. The type and dose of immunosuppressant drugs were noted. Fetal ultrasound evaluation was routinely performed in all pregnancies in all trimesters.

Maternal and obstetric complications during gestation and puerperium were registered. Adverse pregnancy outcome was defined as miscarriage (fetal loss before the 20th week of gestation), preterm delivery (delivery before the 37th week of gestation), and development of hypertensive disorders of pregnancy or perinatal death (stillbirth or neonatal death). 

An exacerbation of AIH was defined as a twofold increase in serum transaminases or appearance of symptoms. Disease remission was defined as an analytic improvement with normalization of serum transaminases levels (16).

Caesarean delivery was performed for obstetrical indications only, except in the presence of cirrhosis. 

## Results

**Patient characteristics**

Seven patients with nine pregnancies were included. Mean age at conception was 31.3 years (23-43 years) and the average time between diagnosis and pregnancy was 8.2 years (2-19 years). All patients had a previous hepatic biopsy to confirm the diagnosis. Two patients had histologically proven cirrhosis before pregnancy. One patient had an overlap syndrome with primary biliary cirrhosis. Before pregnancy, eight women were treated with prednisolone (88.9%) and six (66.7%) with azathioprine. The detailed profile is shown in table 1. 

**Disease activity during pregnancy**

Two patients (22,2%) experienced a flare during pregnancy. One patient showed elevation of hepatic enzymes during the first trimester before a miscarriage. This analytic alteration resolved spontaneously after the pregnancy loss. Another patient experienced a flare at the end of the second trimester, at the 27^th^ week of gestation (table 1 - patient B, pregnancy 4). This flare upThisf was resistant to the increment of medication (increment of prednisolone dosage to 1mg/Kg/day plus azathioprine 100mg/day). No other causes for the analytical exacerbation were found. This allowed the diagnosis of a resistant AIH flare. Termination of pregnancy by caesarean section was performed to control hepatic disease at the 33^th^ week of pregnancy. One patient experienced an analytical improvement after the second trimester (table 1 – patient G, pregnancy 9). The disease remained stable in other pregnancies. There were no clinical or analytical exacerbations during puerperium.

***Obstetrical outcome***

All patients started obstetric evaluation before the 10^th^ week of pregnancy. There were three first trimester miscarriages (33.3% of pregnancies). One patient experienced two first trimester miscarriages, and in the subsequent study of this obstetric complication, a positive result of anti-phospholipid antibodies was found. The third pregnancy of this patient was medicated with acetylsalicylic acid (ASA) 100mg/day and enoxaparin 40mg/d. This patient experienced a clinical aggravation as previously described.

There were no other obstetric complications like hypertensive disorders of pregnancy, foetal growth restriction, stillbirths, foetal malformations or neonatal deaths. We report a caesarean rate of 67% (see table 1 for indications). 

**Table 1:** Pregnancies with autoimmune hepatitis

**Patient**	Pregnancy	Age	Obstetric History	Maternal disease	Medication prior pregnancy	Medication during pregnancy	Pregnancy evolution	Newborn	Post-pregnancy evolution
**A**	1	25	Gravida 2 Para 1+0	AIH type 1 diagnosed in 2000 (4 years before pregnancy) Liver cirrhosis (Child-Pugh A)	Azathioprine 50mg 2id Prednisolone 5mg /day	Azathioprine 50mg 2id Prednisolone 40mg/day Acetylsalicylic acid (ASA) 100mg/day	Ø complications	Spontaneous labour Vaginal delivery 36 weeks 2800g Apgar Index 9/10 1^st^ minute/5^th^ minute	Ø complications
**B**	2	25	Gravida 1 Para 0	AIH type 1 diagnosed in 2000 (9 years before pregnancy) Thalassemia Minor	Azathioprine 50mg 2id Prednisolone 7,5mg/day	Azathioprine 50mg 2id Prednisolone 7,5mg/day	First trimester miscarriage	-	Ø complications
	3	28	Gravida 2 Para 0+1	AIH type 1 diagnosed in 2000 (10 year before pregnancy) Thalassemia Minor	Prednisolone 7,5mg/day	Prednisolone 7,5mg/day ASA 100mg/day	First trimester miscarriage	-	Ø complications
	4	29	Gravida 3 Para 0+2	AIH type 1 diagnosed in 2000 (10 year before pregnancy) Thalassemia Minor Positivity for antiphospholipid antibodies)	Prednisolone 7,5mg/day Hydroxychloroquine 400mg/day	Prednisolone 7,5mg/day (dose increment at the end of second trimester up to 60 mg/day) Hydroxychloroquine 400mg/day Enoxaparin 40mg/day Azathioprine 50mg 2x/day (beginning at 27^th^ week) Acetylsalicylic acid 100mg/day	Analytical and clinical exacerbation, beginning at 27^th^ week of pregnancy	Elective caesarean at form maternal reason 33th week of pregnancy 1950g Apgar Index 8/9 1^st^ minute/5^th^ minute	Good clinical and analytical evolution during the first weeks postpartum
**C**	5	43	Gravida 4 Para3+0	AIH type 1 diagnosed in 2007(2 year before pregnancy)	Prednisolone 7,5mg/day Ursodeoxycholic acid 250mg 3id	Prednisolone 7,5mg/day Ursodeoxycholic acid 250mg 3id	Ø complications	Urgent caesarean at 38th week of pregnancy, secondary to foetal distress (true umbilical knot) 3065g Apgar Index 2/7 1^st^ minute/5^th^ minute	Ø complications
**D**	6	27	Gravida 1 Para 0	AIH type 1 diagnosed in 1992 (19 year before pregnancy) Liver cirrhosis with portal hypertension (Child-Pugh A)	Azathioprine 50mg 2id Propranolol 40mg 3xid Prednisolone 7,5mg/day	Azathioprine 50mg 2id Propranolol 40mg 3xid Prednisolone 7,5mg/day	Ø complications	Urgent caesarean at 38th week of pregnancy, secondary to fetal distress after spontaneous membrane rupture 2550g Apgar Index 7/8 1^st^ minute/5^th^ minute	Ø complications
**E**	7	34	Gravida 1 Para 0	AIH type 3 diagnosed in 2001 (12 year before pregnancy)	Azathioprine 50mg 2id Prednisolone 5mg/day	Azathioprine 50mg 2id Prednisolone 5mg/day	First trimester miscarriage Analytical exacerbation 2 weeks before miscarriage	-	Good clinical and analytical evolution during the first weeks after miscarriage
**F**	8	38	Gravida 5 Para 3+1	AIH type 1 diagnosed in 2009 (3 years before pregnancy)	Azathioprine 50mg 2id	Azathioprine 50mg 2id	Ø complications	Elective caesarean at 38th week of pregnancy, pelvic presentation 3618g /Apgar Index 8/9 1^st^ minute/5^th^ minute	Ø complications
**G**	9	33	Gravida 1 Para 0	AIH type 1 diagnosed in 2009 (5 years before pregnancy)	Azathioprine 50mg 2id Prednisolone 5mg/day	Azathioprine 50mg 2id Prednisolone 5mg/day	Analytical improvement during the second trimester	Vaginal delivery 3750g /Apgar Index 9/10 1^st^ minute/5^th^ minute	Ø complications

## Discussion

Autoimmune hepatitis is a rare cause of chronic hepatic disease in Portugal (17). Therefore, the association between AIH and pregnancy is rare in our country.

We report a high rate of first trimester miscarriage (33.3%), which was also described previously by Candia, et al. (18), Schramm, et al. (9) and Terrabuio et al (7). In our study, two miscarriages occurred in the same patient, and during the investigation of recurrent abortions, a positive title for antiphospholipid antibodies was found. The association between antiphospholipid antibodies and AIH is not frequently found, especially during pregnancy (19). This patient achieved a viable pregnancy under treatment for antiphospholipid antibodies syndrome. 

We also report a preterm delivery rate of 33.3% with one case being iatrogenic, secondary to a severe hepatic flare, and one case spontaneous. There were no hypertensive disorders of pregnancy, maternal deaths or hepatic failure in our group of patients. 

A high rate of caesarean delivery was found in this group of patients. The way of delivery should be chosen by obstetric indication. However, some authors consider that the rise in the abdominal pressure in the presence of hepatic cirrhosis, increase portal hypertension and the risk of bleeding. In our group of patients, caesarean sections were all made for obstetric indications.

We report 2 cases of hepatic flare during pregnancy (22.2%). However, unlike the previous reports, we didn’t find any exacerbation during the postpartum period (7-11). In our institution, we usually increase the corticosteroid dosage during the postpartum to reduce the probability of a flare, as supported by the same author (20), which could explain the absence of AIH flares during the postpartum period. One flare was controlled after a first-trimester miscarriage without increment of medication dosage, and the second case was refractory to the corticosteroid increment of dosage. Therefore, the pregnancy was interrupted at the 33^rd^ week of gestation. The usage of azathioprine during pregnancy has been considered secure (21-24), and all our kept this drug during pregnancy. No cases of foetal malformations were detected in this group of patients. 

The good obstetric outcome could be expected in patients with AIH. However, a tight surveillance by a trained multidisciplinary team is mandatory to allow early diagnose and treatment of AIH flares, reducing the ominous complications described in the first series of pregnancies in these patients.
